# COVID-19 pandemic impact on hypertension management in North East London: an observational cohort study using electronic health records

**DOI:** 10.1136/bmjopen-2023-083497

**Published:** 2024-08-06

**Authors:** Stuart Christopher Gorthorn Rison, Oliver C Redfern, Rohini Mathur, Isabel Dostal, Chris Carvalho, Zahra Raisi-Estabragh, John Robson

**Affiliations:** 1 Clinical Effectiveness Group, Centre for Primary Care, Wolfson Institute of Population Health, Queen Mary University of London, London, UK; 2 Integrated Care System, NHS North East London, London, UK; 3 Nuffield Department of Clinical Neurosciences, Oxford University, Oxford, UK; 4 Saint Bartholomew's Hospital Barts Heart Centre, London, UK; 5 Queen Mary University of London William Harvey Research Institute, London, UK

**Keywords:** Hypertension, Blood Pressure, Health Equity, Primary Care, Cardiovascular Disease, PUBLIC HEALTH

## Abstract

**ABSTRACT:**

**Objective:**

There are established inequities in the monitoring and management of hypertension in England. The COVID-19 pandemic had a major impact on primary care management of long-term conditions such as hypertension. This study investigated the possible disproportionate impact of the pandemic across patient groups.

**Design:**

Open cohort of people with diagnosed hypertension.

**Settings:**

North East London primary care practices from January 2019 to October 2022.

**Participants:**

All 224 329 adults with hypertension registered in 193 primary care practices.

**Outcomes:**

Monitoring and management of hypertension were assessed using two indicators: (i) blood pressure recorded within 1 year of the index date and (ii) blood pressure control to national clinical practice guidelines.

**Results:**

The proportion of patients with a contemporaneous blood pressure recording fell from a 91% pre-pandemic peak to 62% at the end of the pandemic lockdown and improved to 77% by the end of the study. This was paralleled by the proportion of individuals with controlled hypertension which fell from a 73% pre-pandemic peak to 50% at the end of the pandemic lockdown and improved to 60% by the end of the study. However, when excluding patients without a recent blood pressure recording, the proportions of patients with controlled hypertension increased to 81%, 80% and 78% respectively.

Throughout the study, in comparison to the White ethnic group, the Black ethnic group was less likely to achieve adequate blood pressure control (ORs 0.81 (95% CI 0.78 to 0.85, p<0.001) to 0.87 (95% CI 0.84 to 0.91, p<0.001)). Conversely, the Asian ethnic group was more likely to have controlled blood pressure (ORs 1.09 (95% CI 1.05 to 1.14, p<0.001) to 1.28 (95% CI 1.23 to 1.32, p<0.001)). Men, younger individuals, more affluent individuals, individuals with unknown or unrecorded ethnicity or those untreated were also less likely to have blood pressure control to target throughout the study.

**Conclusion:**

The COVID-19 pandemic had a greater impact on blood pressure recording than on blood pressure control. Inequities in blood pressure control persisted during the pandemic and remain outstanding.

**Strengths and limitations of this study**

- The study involved a large and unselected cohort in a socially diverse population with high levels of recorded self-reported ethnicity.
- The study included 12 months of pre-pandemic data and over 12 months of post-lockdown data.
- A blood pressure recording initiative launched within a subset of the study practices permitted an analysis of the impact of up-to-date blood pressure recordings on the study outcomes.
- The study could not account for the impact of the pandemic-related fall in new hypertension diagnoses on the outcomes.
- Factors not included in the study may account for some of the observed outcome variations.

## Introduction

 The COVID-19 pandemic has had a profound impact on the provision of primary care services and the management of long-term conditions. Service provision in the UK, and internationally, transitioned from largely face-to-face primary care appointment models to remote-consultation models reducing opportunities to directly access care.^1^,^3^ These changes in healthcare delivery were observed at multiple levels including monitoring of long-term conditions,^4 5^ medication prescribing^6 7^ and diagnosing new conditions.^8^,^10^

The SARS-CoV-2 virus also disproportionately affected certain demographic groups both in terms of risk of infection and rate of mortality.^11^,^13^ COVID-19 was associated with excess mortality in those of older age and with higher levels of deprivation and highest in Black and South Asian ethnic groups; this was most marked in those with multimorbidity including cardiovascular disease, severe obesity and impaired renal function.^12^

Primary care management of pre-existing long-term conditions such as hypertension also worsened and may have disproportionately affected certain ethnic or other social groups.^7 14 15^ Our study develops a previous study of pre-pandemic hypertension management inequities^16^ by following an ethnically diverse cohort of individuals with hypertension monitored over 46 months spanning the pre-pandemic, pandemic lockdown and pandemic recovery phases of the COVID-19 outbreak. The objectives were to assess the impact of the COVID-19 pandemic on management of blood pressure of adult patients with hypertension in North East London and to identify any health inequities in the impact of COVID-19 on the cohort with respect to reported ethnicity, sex, age, socioeconomic status and treatment intensity.

## Methods

### Study Population

The study was carried out in five contiguous North East London localities: City and Hackney, Newham, Redbridge, Tower Hamlets and Waltham Forest with a total adult population of approximately 1.4 million individuals. The study included 193 general practices which used the EMIS electronic health record system (EMIS health, Leeds, UK) excluding the remaining six practices which used a different electronic health record system and were not accessible to the researchers.

Pseudonymised individual-level data were collected for all adults registered with a general practitioner (GP) practice between January 2019 and October 2022. Each month, the cohort comprised currently registered adults aged 18 years and older with an extant diagnosis of hypertension on the first of the month (the index date for that month’s cohort data). The English national Quality and Outcomes Framework code set identified hypertension excluding ‘hypertension resolved’ (online supplemental table S1 and https://clinicalcodes.rss.mhs.man.ac.uk/medcodes/article/203/).^16 17^

### Demographic Variables

For each individual at each monthly index date, the following demographic data were extracted (online supplemental table S2): age in years on index date, sex, ethnicity code, study locality and location of residence as defined by Lower Layer Super Output Area (LSOA) (a geographic division for reporting of small area statistics in England and Wales^18^).

Age was categorised into one of the following bands: 18–29 years, then to 10-year age bands; 30–39 years, 40–49 years, to 90 years and above.

To account for socioeconomic status, the Office of National Statistic’s Index of Multiple Deprivation (IMD) 2019 was used.^19^ The IMD is the official measure of small area deprivation in England; seven distinct domains are considered (eg, income, housing, crime) to generate a single IMD score for each LSOA.^18 19^ IMD scores were used to categorise individuals into a national IMD quintile; IMD quintile 1 (most deprived) to quintile 5 (least deprived).

Ethnicity codes were categorised according to the Office of National Statistics 2001 census categories and comprised White (including White British, Irish or White other), Black (including Black British, Caribbean, African and other Black background), Asian (including British Asian, Bangladeshi, Pakistani, Indian and any other Asian background), Chinese and other ethnic groups (classified as Other ethnic groups) and Mixed ethnicity.^20^ The Unknown ethnic group comprised individuals with no ethnicity code recorded, individuals with unclassifiable ethnicity codes and individuals with a ‘not stated’ code.

### Clinical variables

The most recent systolic blood pressure (SBP) and diastolic blood pressure (DBP) values (in mm Hg) and their entry dates were extracted on each index date. Blood pressure (BP) recordings were excluded if dating from more than 1 year prior to the index date. Recordings were also excluded if they were incomplete, unreliable or contained implausible blood pressures, that is, SBP but no DBP recorded (or vice versa), separately recorded blood pressure elements (SBP date different from DBP date) and SBP <70 mm Hg or SBP >=270 mm Hg or DBP <40 mm Hg or DBP >=150 mm Hg.

In England, control of blood pressure in individuals with hypertension is a nationally audited metric as part of the financially incentivised national Quality and Outcomes Framework (QOF).^21^ Therefore, blood pressure control was defined by QOF indicators; that is, for individuals under 80 years of age as a systolic blood pressure less than or equal to 140 mm Hg and diastolic blood pressure less than or equal to 90 mm Hg; for individuals 80 years of age and older as a systolic blood pressure less than or equal to 150 mm Hg and a diastolic blood pressure less than or equal to 90 mm Hg. All measures were to have been recorded within the preceding 12 months and used the total register of diagnosed hypertension as the denominator so that those with blood pressure ‘not recorded in 12 months’ were effectively considered as not controlled.^21^

Medicines prescribed in the 6 months up to and including each index date were considered for eight classes of antihypertensive medication (online supplemental table S2): (i) ACE inhibitors/angiotensin receptor blockers, (ii) beta-blockers, (iii) potassium-sparing diuretics, (iv) calcium channel blockers, (v) thiazide-type and thiazide-like diuretics, (vi) centrally acting antihypertensives, (vii) alpha-blockers and (viii) loop diuretics. The number of different medication classes prescribed was used to define three categorical treatment intensities, that is, individuals on zero, a single, or two or more antihypertensive medication classes.^16^

The 1 April 2019, 2020, 2021 and 2022 were the representative index dates for the following pandemic-related phases: pre-pandemic, pandemic pre-lockdown, pandemic lockdown and pandemic recovery respectively.^22^ These phases defined the study phase categorical variable our models.

### Outcomes

Three binary outcome variables of blood pressure management were considered:

1. BLOOD PRESSURE RECORDED: A valid blood pressure recorded within 12 months of the index date.
2. QOF BP CONTROL: A blood pressure within the QOF age-adjusted target. In keeping with the QOF hypertension indicators criteria, all individuals *without* a blood pressure recorded within the 12 months to the index date were considered to have uncontrolled blood pressure.
3. RECORDED BP CONTROL: A blood pressure recorded within 12 months leading up to and including the index date within the QOF age-adjusted blood pressure targets.

The difference between the QOF BP CONTROL and the RECORDED BP CONTROL outcomes becomes apparent when considering them at cohort level. When calculating the percentage of individuals with QOF BP CONTROL and RECORDED BP CONTROL, the numerator is the same for both indicators (individuals with a valid blood pressure most recently recorded in 12 months to the index date were within the QOF age-adjusted target). However, for QOF BP CONTROL, the denominator is the total number of individuals in the cohort, whereas, for RECORDED BP CONTROL, the denominator is only the number of individuals with a blood pressure recorded in 12 months to the index date.

### Statistical methods

Data were processed, aggregated and validated, and descriptive statistics for all demographic, clinical and outcome variables were calculated. The percentages of patients meeting each outcome criteria were plotted at for each of the study’s 46 monthly time points to generate outcome trajectories. Correlations between outcome trajectories were measured using Kendall’s rank correlation coefficient (Kendall’s τ).

Unadjusted and adjusted (for ethnic group, sex, age, IMD quintile, pandemic phase and treatment intensity) multivariable logistic regression analyses were completed for each outcome indicator. The analyses for the RECORDED BP CONTROL outcome were performed on the subset of individuals with a valid blood pressure recorded within 12 months to the index date.

During the study, the impact of COVID-19 lockdowns led the City and Hackney locality to launch a blood pressure recording initiative in May 2020.^23^ To assess the impact of this intervention, a subanalysis comparing localities was performed.

All analyses were performed using Python (V.3.9.1) and R (V.4.0.5). Forest plots were generated using the forestplot Python package (V.0.2.0).^24^

### Patient and public involvement

The research was designed and conducted without patient or public involvement.

## Results

### Study cohort and demographic and clinical variables

**Figure 1:**
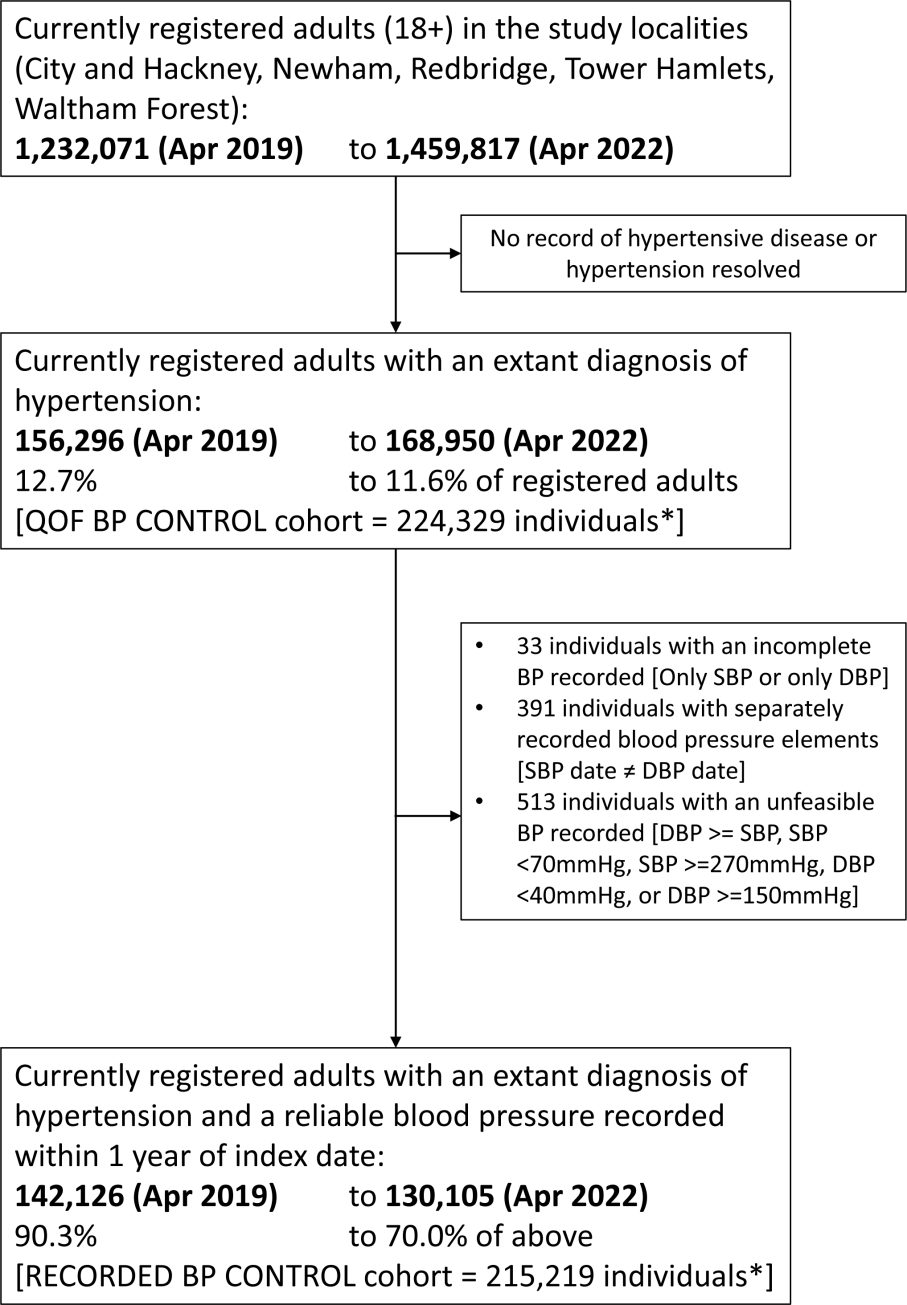
Study open cohort flowchart. The study ran from 1 January 2019 to 1 October 2022 with April 2019 and April 2022 representing the pre-pandemic and post-lockdown cohort respectively. *Unique cohort members over the whole study period. BP, blood pressure; DBP, diastolic blood pressure; SBP, systolic blood pressure.

Over the 46 months of the study, data from 224 329 individuals were considered, with an average of 33.5 patient months of follow-up (figure 1 and table 1). 113 255/224 329 [50.5%] of individuals were present throughout the 46 months of the study. 215 219/224 329 [95.9%] of individuals had at least one valid blood pressure recording throughout the study period (online supplemental table S3).

**Table 1:** Characteristics of patients included in the analysis

Individual patients (n)	224 329	
Age (years): mean (SD)	61.3 (14.6)	
Systolic BP (mm Hg): mean (SD)	133.6 (14.3)	
Diastolic BP (mm Hg): mean (SD)	79.0 (10.2)	
Time in study (months): mean (SD; min.; max.; mode)	33.5 (15.7; 1; 46; 46)	
Sex: n (%)		
**Female**	**113 604(50.6%)**	
Male	110 725 (49.4%)	
Ethnicity: n (%)		
**White**	**82 624(36.8%)**	
Asian or Asian British	68 701 (30.6%)	
Black or Black British	46 111 (20.6%)	
Unknown	12 883 (5.7%)	
No ethnicity code recorded	7872 (3.5%)	
Unclassified code	3504 (1.6%)	
‘Not stated’ code	1507 (0.7%)	
Other ethnic group	9255 (4.1%)	
Mixed	4755 (2.1%)	
Age distribution: n (%)		
(18–30)	2578 (1.1%)	
(30–40)	12 764 (5.7%)	
(40–50)	33 333 (14.9%)	
**(50–60)**	**55 856(24.9%)**	
(60–70)	53 850 (24.0%)	
(70–80)	38 119 (17.0%)	
(80–90)	22 802 (10.2%)	
(90–120)	5027 (2.2%)	
Index of Multiple Deprivation (IMD) quintile: n (%)		
Q1 (most deprived)	59 368 (26.5%)	
**Q2**	**104 568(46.6%)**	
Q3	36 766 (16.4%)	
Q4	16 857 (7.5%)	
Q5 (least deprived)	6634 (3.0%)	
Q0 (unrecorded)	136 (0.1%)	
Number of antihypertensive medications: n (%)		
0	39 591 (17.6%)	
**1**	**82 642(36.8%)**	
2	61 305 (27.3%)	2+:102 096(45.5%)
3	28 354 (12.6%)	
4	9659 (4.3%)	
5+	2778 (1.2%)	

Characteristics are derived from the first presentation of an individual in the open cohort except for time in study which considers individuals throughout the study period (n=224 329 adults with a diagnosis of hypertension). For categorical characteristics, the most common category is shown in bold.

BPblood pressureSDstandard deviation

Characteristics of the cohort are described in table 1. 96.5% of individuals had a self-reported ethnicity code recorded and an Office of National Statistics ethnic group was assigned to 94.3% of individuals; 5.7% had no ethnicity code, ‘not stated’ ethnicity codes or ethnicity codes which could not be mapped to an Office for National Statistics ethnic group.

The cohort make-up in terms of ethnic groups, ages, sex and IMD quintiles remained largely unchanged over the study phases (online supplemental figure S1).

### Outcomes

**Figure 2:**
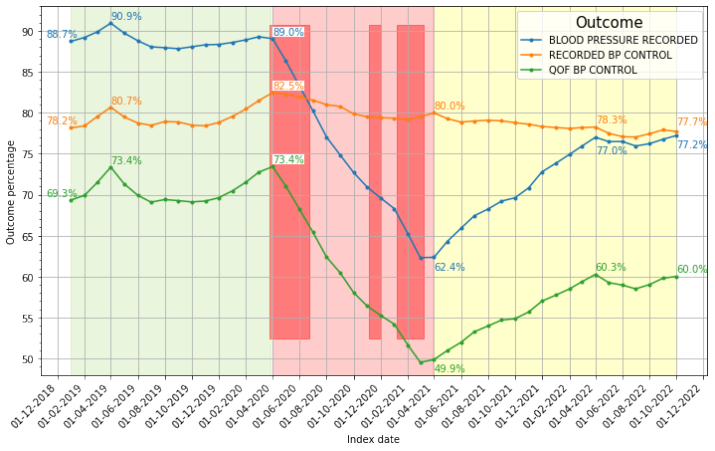
Percentage of cohort individuals meeting the BLOOD PRESSURE RECORDED, RECORDED BP CONTROL and QOF BP CONTROL outcomes over the course of the study. Pre-pandemic time points are shown in the green-shaded area, pandemic-lockdown time points in the light red area and pandemic-recovery time points in the yellow-shaded area. The three darker red rectangles represent the three England-wide COVID-19 lockdowns. Percentages are printed for the April 2019, 2020, 2021 and 2022 outcomes as well as for the first and last months of the study.

The outcome variables were plotted as cohort percentages monthly over the study period (figure 2). Blood pressure recordings (BLOOD PRESSURE RECORDED) showed a large fall during the pandemic lockdown phase from 89% in April 2020 to 62% at the end of the lockdown phase, paralleled by a fall in QOF BP CONTROL from 73% to 50%. Both indicators then showed a marked rise in the pandemic recovery phase but not to pre-pandemic levels. The BLOOD PRESSURE RECORDED and QOF BP CONTROL outcomes were highly correlated (Kendall’s τ=0.90; p value <0.001). In contrast, the RECORDED BP CONTROL indicator exhibited a smaller fall during lockdown, from a peak of 83% in April 2020 to 78% at the end of the study. Furthermore, there was no significant correlation between the RECORDED BP CONTROL and BLOOD PRESSURE RECORDED outcomes (Kendall’s τ=0.05; p value 0.59).

### Impact of local blood-pressure recording initiative

The impact of the local blood pressure recording initiative is illustrated in online supplemental figure S2 which plots outcomes by locality. Following the launch of the initiative in the City and Hackney intervention locality in May 2020, the BLOOD PRESSURE RECORDED remained at around 76% from July 2020 for the lockdown period of the study. In contrast, in other localities, BLOOD PRESSURE RECORDED continued to fall to a nadir of approximately 60% at the end of the lockdown period. For all localities, the QOF BP CONTROL closely paralleled the BLOOD PRESSURE RECORDED: for the City and Hackney intervention locality, Kendall’s τ was 0.71 (p value <0.001) and for the combined non-intervention localities, Kendall’s τ was 0.89 (p value <0.001). However, there was no strong association between the BLOOD PRESSURE RECORDED and RECORDED BP CONTROL outcomes (City and Hackney: Kendall’s τ=0.35; p value <0.001; non-intervention localities: Kendall’s τ=−0.14; p value 0.18); that is, increasing the percentage of individuals with a recently recorded blood pressure did not results in a matched increase in the percentage of patient with a recorded blood pressure meeting the QOF blood pressure control target.

### Study phases

**Figure 3:**
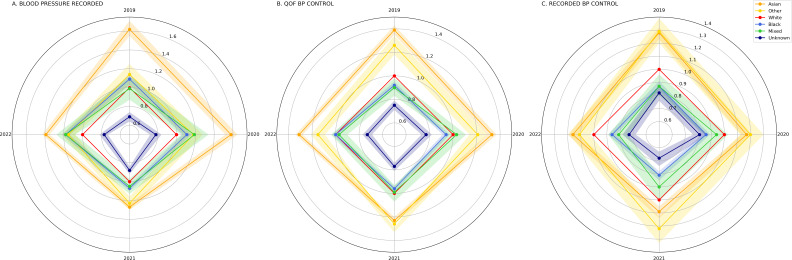
Radar charts of study outcome OR (clockwise from 12 o’clock: April 2019 (pre-pandemic), April 2020 (pre-lockdown), April 2021 (lockdown), April 2022 (recovery)) by study ethnic group: (A) BLOOD PRESSURE RECORDED. (B) QOF BP CONTROL. (C) RECORDED BP CONTROL. 95% CI are shown (shaded areas). The White ethnic group (red line) is the reference group and therefore, plotted with an OR of 1.0 and no CI.

For each outcome, the OR for the different ethnic groups derived from the multivariate models at each of the four study-phase annual index dates were plotted on a radar plot chart (figure 3). Forest plots of OR, including 95% CIs and p values, per ethnic group are shown in online supplemental figure S3. This simplified representation in figure 3 allows the reader to identify patterns of outcome variation over the study period—for example, relative to the White ethnic group and regardless of the pandemic phase, the Black ethnic group always had a lower likelihood (lower OR) of QOF BP CONTROL and RECORDED BP CONTROL (ie, the Black ethnic group polygon (light blue) is fully circumscribed by the White ethnic group polygon (red)). Conversely, the Asian ethnic group always had higher OR than the White ethnic group. However, relative to the White ethnic group, the Black ethnic group was similarly or more likely to have a recent blood pressure recorded (BLOOD PRESSURE RECORDED, figure 3 and online supplemental figure S3).

The Mixed-ethnicity ethnic group closely paralleled the Black ethnic group for the three study outcomes (ie, more likely to have a blood pressure recorded than the White ethnic group but less likely to have controlled hypertension, figure 3 and online supplemental figure S3). Individuals of unknown ethnicity always had the lowest ORs of all ethnic groups for all study outcomes (figure 3 and online supplemental figure S3).

The whole-model results are given in online supplemental figure S4. The salient impact of treatment intensity is summarised in online supplemental table S4.

## Discussion

### Summary

This study followed a large open cohort of 224 329 adults with hypertension from all eligible 193 general practices in North East London over 46 months including the pre-pandemic, the pandemic pre-lockdown, the pandemic lockdown and the pandemic recovery phases illustrating its impact on three indicators of hypertension management. More than 1 year after the pandemic lockdowns, levels of control of blood pressure had improved but had not been restored to pre-pandemic maxima (figure 2). At the start of the study (January 2019), RECORDED BP CONTROL was 78.2%, by the end of the study (October 2022), it was 77.7%. However, this before and after comparison conceals a larger more recent deterioration as the pre-lockdown peak was 82.5% in April 2020.

Because of low rates of blood pressure recording, the national QOF outcome for hypertension control QOF BP CONTROL yielded misleadingly low estimates as it conflated lack of recording with lack of control.^25 26^ This was highlighted by the City and Hackney lockdown initiative which effected a notable improvement in the proportion of blood pressure recorded within a year, and consequently, in the QOF-derived QOF BP CONTROL outcome but had little impact on the control of blood pressure in those in whom blood pressure was recorded (RECORDED BP CONTROL outcome) (online supplemental figure S3).^23^

In other words, the pandemic impacted all indicators of hypertension management, but the impact on recording of blood pressure was much more marked than reductions in the control of blood pressure.

Pre-pandemic ethnic group outcome variations^16^ persisted in all pandemic phases. Both before (April 2019), and during (April 2020 and April 2021) and after the pandemic lockdown (April 2022), the Black ethnic group was less likely to have controlled hypertension (RECORDED BP CONTROL ORs 0.87 (2019), 0.86 (2020), 0.81 (2021) and 0.86 (2022), all p values <0.001), and the Asian ethnic group more likely to have controlled hypertension (RECORDED BP CONTROL ORs 1.28 (2019), 1.17 (2020), 1.09 (2021) and 1.16 (2022), all p values <0.001), than the White ethnic group (figure 3 and online supplemental figure S3). Throughout the study, individuals in the unknown ethnicity group were the least likely of all to have blood pressures recorded (BLOOD PRESSURE RECORDED ORs 0.69 (2019), 0.78 (2020), 0.88 (2021) and 0.77 (2022), all p values <0.001) or managed to target (RECORDED BP CONTROL ORs 0.82 (2019), 0.81 (2020), 0.68 (2021) and 0.73 (2022), all p values <0.001), representing a relatively small (6% of the cohort) but high-risk group.

Older adults and individuals living in more deprived areas were more likely to have controlled hypertension than younger patients and individuals in more affluent areas. Control of hypertension was also less likely in men than women throughout the study period.

### Impact of treatment intensity

The full model data highlighted the marked relationship between treatment intensity and the likelihood of having a blood pressure recorded within 12 months (online supplemental figure S4). For the BLOOD PRESSURE RECORDED outcome, relative to untreated individuals, individuals on one antihypertensive had ORs ranging from 3.27 (2021) to 7.83 (2019) (all p values <0.001; online supplemental table S4). Individuals on two or more antihypertensives had ORs ranging 4.1 (2021) to 9.99 (2019) (all p values <0.001; online supplemental table S4). With respect to the control of hypertension outcomes (QOF BP CONTROL and RECORDED BP CONTROL), treated individuals were unsurprisingly more likely to have controlled hypertension than untreated individuals but there was little difference in the ORs for individuals on a single, or two or more antihypertensives (online supplemental table S4).

Untreated individuals represent a doubly at-risk group, more likely to have uncontrolled hypertension and less likely to have this recognised by their healthcare team but accounting for approximately one in six of the individuals in the study cohort (table 1). Untreated individuals were more likely to be younger, male, of unknown ethnicity, perhaps representing patients who may find access to primary care difficult or be less willing to engage with healthcare providers.

Understanding systemic and patient-level obstacles to blood pressure recording effective treatment may identify obstacles such as prescription charges or easier access to recording facilities including community pharmacies or more flexible GP appointments outside normal working hours.^27^,^29^

### Comparison with existing literature

The ethnic group inequities identified in this study have been identified in other UK pre-pandemic studies which also found a higher likelihood of blood pressure control in the Asian ethnic group and a lower likelihood of blood pressure control in the Black ethnic group compared with the White ethnic group.^30^,^33^

Comparing the 2020 and 2021 England National Cardiovascular Disease Prevention Audits, the authors reported similar findings. The pandemic caused a similar reduction across sex, age and ethnic group in the national recordings of blood pressure.^26^ In those with recent blood pressure recorded, there was little change in blood pressure control by age, deprivation and ethnic group—an indicator identical to this study’s QOF BP CONTROL outcome. The authors concluded that the pandemic impact was largely on recording of blood pressure rather than control.^26^ Likewise, an analysis of 25.2 million patient records in the OpenSAFELY analytical platform concluded that the disruption to hypertension management QOF indicators during the pandemic could be attributed to a reduction in blood pressure recording.^34^

PCORnet, a large study from 24 US health systems from 2017 to 2020, investigated indicators of hypertension control including the percentage of patients with a blood pressure <140/90 mm Hg.^35^ Findings for this indicator paralleled those in our study: patients of Asian ethnicity were more likely, and patients of Black ethnicity were less likely than patients of White ethnicity to have controlled hypertension, despite higher use of follow-up visits in the Black ethnic group. Weighted average BP control was uncontrolled for 60.5% of individuals in the PCORnet 2019 pre-pandemic cohort and dropped over 7.2 percentage points for the 2020 pandemic cohort. In our study, the equivalent RECORDED BP CONTROL outcome peak to trough was 4.8% (82.5 to 77.7%).

### Limitations

The demographic inequities identified in our study might be due to other confounding factors such as adherence to treatment or ethnic variability in the prevalence of resistant hypertension.^3336^,^40^ However, our findings confirm those of previous studies of ethnicity-related inequities in the management of hypertension.^30^,^33^

While we were able to assign 94% of cohort individuals to one of five defined ethnic groups, these may conceal underlying heterogeneity within categories.^41^

Urban living means that the affluent and more deprived often live in close proximity and measures such as IMD quintile based on small areas may reduce gradients between extremes of deprivation in dense urban areas. In addition, the more affluent individuals may have been able to move away from London during the pandemic lockdown phase or access alternative health services which may further influence blood pressure recording and control and contribute to apparent poor management in this group.

The study may also have been affected by the impact of the pandemic on hypertension case finding; a UK-wide study found that 500 000 fewer people than expected started taking blood pressure-lowering medication between March 2020 and July 2021.^10^

Lastly, although the study cohort was large and diverse, it was geographically limited to North East London and therefore, study findings may not generalise to other parts of England.

## Conclusions

Pre-existing inequities in blood pressure monitoring and control persisted during the pandemic and remain outstanding. In the study cohort, the COVID-19 pandemic had a greater impact on blood pressure recording than on blood pressure control per se.

Metrics of hypertension management which combine aspects of monitoring and control of blood pressure might therefore be prone to misinterpretation. QOF indicators may serve as a suitable ‘performance’ metrics, but a full assessment of hypertension control requires separate consideration of both the recording of blood pressure and adequate control in those recorded.

The COVID-19 pandemic effected a radical change in the provision of primary care services and reduced opportunities for recording of risk factors and optimising management. Our data show that despite substantial improvements, more than a year after the pandemic, recovery to pre-pandemic levels had yet to be achieved. Equitable improvements in the management of hypertension remained an outstanding priority for men, younger individuals, and within the Black and Mixed ethnic groups.^42^ Furthermore, the lack of ethnicity recording may alert clinicians to individuals at a higher risk of suboptimal care.

The data that support the findings of this study are not openly available due to reasons of data governance.

The code sets used in this study are available from https://clinicalcodes.rss.mhs.man.ac.uk/medcodes/article/203/.

## supplementary material

10.1136/bmjopen-2023-083497 online supplemental file 1

## Data Availability

No data are available.
