# Supplementary material for: COVID-19 pandemic impact on hypertension management in North East London: an observational cohort study using electronic health records
Source: BMJ Open. 2024 Aug 6;14(8):e083497. doi: 10.1136/bmjopen-2023-083497 (PMC11308888; doi:10.1136/bmjopen-2023-083497)
Supplement: online supplemental file 1 [file bmjopen-14-8-s001.pdf]

Supplementary material for: COVID-19 pandemic impact on hypertension management in North-East London: an observational cohort study using electronic health records.

AUTHORS

- Stuart CG Rison[1,2] (ORCID: 0000-0003-3289-6668), PhD, MBBS, GP and clinical researcher
- Oliver C Redfern[3] (ORCID: 0000-0002-3596-3806), PhD, MBBS, Clinical researcher
- Rohini Mathur[1] (ORCID: 0000-0002-3817-8790), BSc, MSc, PhD, Professor and Chair of Health Data Science
- Isabel Dostal[1] (ORCID: 0009-0007-8896-3010) BSc, MSc, Research assistant
- Chris Carvalho[1,2] (ORCID: 0000-0002-4114-9210), MBChB, MSc, GP and clinical research fellow
- Zahra Raisi-Estabragh[4,5] (ORCID: 0000-0002-7757-5465), MBChB, PhD, NIHR Academic Clinical Lecturer in Cardiology
- John P Robson[1] (ORCID: 0000-0001-6889-0415), MD, Reader in Primary Care Research and Development

AFFILIATIONS

1. Clinical Effectiveness Group, Centre for Primary Care, Wolfson Institute of Population Health, Barts and The London School of Medicine and Dentistry, Queen Mary University of London, London, UK. j.robson@qmul.ac.uk
2. North East London Integrated Care System, Unex Tower, London, UK.
3. Nuffield Department of Clinical Neurosciences, University of Oxford, Oxford, UK
4. Barts Heart Centre, St Bartholomew's Hospital, Barts Health NHS Trust, London, UK
5. William Harvey Research Institute, National Institute for Health and Care Research Barts Biomedical Research Centre, Queen Mary University London, London, UK.

Corresponding author: Stuart CG Rison, s.rison@qmul.ac.uk

SUPPLEMENTARY MATERIAL

Supplementary Tables

Hypertension Code	Coding System	Description	Associated Read Code(s)
G2	Read	Hypertensive disease	
G2_	Read	Hypertensive disease	
G20	Read	Essential hypertension	
G20..	Read	Essential hypertension	
G24	Read	Secondary hypertension	
G24.. (excl. G24z1)	Read	Secondary hypertension	
G25	Read	Stage 1 hypertension (NICE - Nat Ins for Hth Clin Excl 2011)	
G25..	Read	Stage 1 hypertension (NICE - Nat Ins for Hth Clin Excl 2011)	
G26	Read	Severe hypertension (Nat Inst for Health Clinical Ex 2011)	
G28	Read	Stage 2 hypertension (NICE - Nat Ins for Hth Clin Excl 2011)	
G2y	Read	Other specified hypertensive disease	
G2y..	Read	Other specified hypertensive disease	
G2z	Read	Hypertensive disease NOS	
Gyu2	Read	[X]Hypertensive diseases	
Gyu2..	Read	[X]Hypertensive diseases	
10441000006116	SNOMED	Other specified hypertensive disease	
1201005	SNOMED	Benign essential hypertension	G20..
1215744012	SNOMED	Hypertensive disorder	
121910014	SNOMED	Benign secondary renovascular hypertension	
131046010	SNOMED	Malignant essential hypertension	
147988014	SNOMED	Malignant secondary hypertension	
151171000006110	SNOMED	Secondary malignant hypertension NOS	
1806071000006102	SNOMED	Stage 1 hypertension	G2y..
1806071000006118	SNOMED	Stage 1 hypertension	
1806081000006104	SNOMED	Stage 2 hypertension	G2y..
1806081000006115	SNOMED	Stage 2 hypertension	
1806141000006109	SNOMED	Severe hypertension	G2y..
1806141000006113	SNOMED	Severe hypertension	
194783001	SNOMED	Secondary malignant renovascular hypertension	G24..
194785008	SNOMED	Secondary benign hypertension	G24..
194788005	SNOMED	Hypertension secondary to endocrine disorders	G24..
196353013	SNOMED	Elevated blood pressure	
196354019	SNOMED	Finding of increased blood pressure	
2164904016	SNOMED	HTN - Hypertension	

2189411000000111	SNOMED	Stage 1 hypertension (NICE - National Institute for Health and Clinical Excellence 2011)	
2189451000000110	SNOMED	Severe hypertension (NICE - National Institute for Health and Clinical Excellence 2011)	
2193021000000110	SNOMED	Severe hypertension	
2193031000000112	SNOMED	Stage 1 hypertension	
2194941000000119	SNOMED	Stage 2 hypertension (NICE - National Institute for Health and Clinical Excellence 2011)	
2211211000000110	SNOMED	Stage 2 hypertension	
2335761000000116	SNOMED	Stage 1 hypertension (NICE 2011) without evidence of end organ damage	
2335801000000114	SNOMED	Stage 1 hypertension (NICE 2011) with evidence of end organ damage	
24184005	SNOMED	Finding of increased blood pressure	G20..
2470030014	SNOMED	Malignant secondary renovascular hypertension	
2470031013	SNOMED	Benign secondary hypertension	
2478822013	SNOMED	Secondary benign renovascular hypertension	
2532161014	SNOMED	Hypertension secondary to endocrine disorder	
2671386015	SNOMED	Hypertensive disorder; systemic arterial	
2920698012	SNOMED	Blood pressure elevation	
299676019	SNOMED	Secondary malignant renovascular hypertension	
299678018	SNOMED	Secondary benign hypertension	
299681011	SNOMED	Hypertension secondary to endocrine disorders	
3135013	SNOMED	Benign essential hypertension	
31992008	SNOMED	Secondary hypertension	G24.., Gyu2..
3763241013	SNOMED	Endocrine hypertension	
38341003	SNOMED	Hypertensive disease	G2_, G20.., G2y.., G2z, Gyu2..
389331000006110	SNOMED	[X]Hypertension secondary to other renal disorders	
389341000006117	SNOMED	[X]Hypertensive diseases	
413461000006118	SNOMED	[X]Other secondary hypertension	
48146000	SNOMED	Diastolic hypertension	G20..
490277011	SNOMED	BP - High blood pressure	
490278018	SNOMED	Systemic arterial hypertension	
490280012	SNOMED	HBP - High blood pressure	
490281011	SNOMED	HT - Hypertension	
490282016	SNOMED	High blood pressure disorder	
490283014	SNOMED	BP+ - Hypertension	
503982017	SNOMED	Accelerated essential hypertension	
508416019	SNOMED	Accelerated secondary hypertension	
523801000006119	SNOMED	BP - hypertensive disease	
53452019	SNOMED	Secondary hypertension	

56218007	SNOMED	Systolic hypertension	G20..
59621000	SNOMED	Essential hypertension	G20..
64168014	SNOMED	Hypertensive disease	
64172013	SNOMED	High blood pressure	
64173015	SNOMED	Hypertensive vascular disease	
64174014	SNOMED	Hypertensive vascular degeneration	
64176011	SNOMED	Hypertension	
648911000006113	SNOMED	Essential hypertension NOS	
73410007	SNOMED	Secondary benign renovascular hypertension	G24..
78975002	SNOMED	Malignant essential hypertension	G20..
80224019	SNOMED	Diastolic hypertension	
843821000000102	SNOMED	Stage 1 hypertension	G25..
843841000000109	SNOMED	Severe hypertension	G26
846371000000103	SNOMED	Stage 2 hypertension	G28
884121000006111	SNOMED	Malignant hypertension	
89242004	SNOMED	Malignant secondary hypertension	G24..
908631000000108	SNOMED	Stage 1 hypertension (NICE 2011) without evidence of end organ damage	G25..
908651000000101	SNOMED	Stage 1 hypertension (NICE 2011) with evidence of end organ damage	G25..
93494011	SNOMED	Systolic hypertension	
99042012	SNOMED	Essential hypertension	
99044013	SNOMED	Idiopathic hypertension	
99046010	SNOMED	Systemic primary arterial hypertension	
99047018	SNOMED	Primary hypertension	
**Hypertension resolved code**	**Coding System**	**Description**	**Associated Read Code(s)**
21261	Read	Hypertension resolved	
212K	Read	Hypertension resolved	
162659009	SNOMED	Hypertension resolved	21261, 212K

Table S1: Hypertension and Hypertension resolved codes. Available from https://clinicalcodes.rss.mhs.man.ac.uk/medcodes/article/203/

Datum	Category
Age	Patient Details
Gender	Patient Details
Lower Layer Area (2011)	Patient Details
Organisation Code	Patient Details
Ethnicity	Clinical Code
Hypertension	Clinical Code
Systolic BP	Clinical Code
Systolic BP	Date
Systolic BP	Value
Diastolic BP	Clinical Code
Diastolic BP	Date
Diastolic BP	Value
ACEi/ARB	Name, Dosage and Quantity
ACEi/ARB	Date of Issue
Beta Blocker	Name, Dosage and Quantity
Beta Blocker	Date of Issue
K-Sparing	Name, Dosage and Quantity
K-Sparing	Date of Issue
CCBs	Name, Dosage and Quantity
CCBs	Date of Issue
Thiazide Diuretic	Name, Dosage and Quantity
Thiazide Diuretic	Date of Issue
Centrally Acting HTs	Name, Dosage and Quantity
Centrally Acting HTs	Date of Issue
Alpha Blockers	Name, Dosage and Quantity
Alpha Blockers	Date of Issue
Loop Diuretic	Name, Dosage and Quantity
Loop Diuretic	Date of Issue

Table S2: Patient data collected.

	Total number of instances	Number of unique cohort individuals
Pre-processing dataset		
Patient months	7,514,995	224,329
Patient blood pressure recordings	*7,286,480*	
Patient instances excluded		
Patients with sex recorded as “Unspecified” or “Unrecorded”	38	[SDL]*
Invalid blood pressure recordings (blood pressure reading excluded but instance not excluded)		
Blood pressure recorded over a year prior to index date	1,406,398	145,526
Incomplete blood pressures (systolic blood pressure but no diastolic blood pressure and vice-versa)	188	[SDL]
Separately recorded blood pressure elements (i.e. date of systolic blood pressure different from date of diastolic blood pressure)	2,240	391
Diastolic blood pressure greater than or equal to systolic blood pressure	343	104
Unfeasible blood pressure (SBP < 70mmHg, SBP ≥ 270mmHg, DBP < 40mmHg or DBP ≥ 150mmHg)	2,348	409
Post-processing dataset		
Patient months	7,514,957	224,329*
Patient months excluded	*38*	
Patient BPs	5,874,925	215,219
Patient BPs excluded	*1,411,555*	

Table S3: Cohort processing. BP = Blood pressure; SBP = Systolic Blood Pressure; DBP = Diastolic Blood Pressure; SDL = statistical disclosure limitation (number in group not disclosed for groups with fewer than 100 individuals). *the overall number of individuals is not affected as these individuals only have Sex recorded as unspecified/unrecorded for a part of the study.

	April 2019 [Pre-pandemic]		April 2020 [Pre-lockdown]		April 2021 [End of lockdown]		April 2022 [Recovery]	
	OR	95% CI	OR	95% CI	OR	95% CI	OR	95% CI
**BLOOD PRESSURE RECORDED**								
1 antihypertensive	7.83	7.48-8.21	6.72	6.44-7.01	3.27	3.17-3.38	4.05	3.93-4.19
2+ antihypertensives	9.99	9.55-10.45	8.57	8.23-8.93	4.10	3.97-4.22	5.18	5.02-5.35
**QOF BP CONTROL**								
1 antihypertensive	3.17	3.07-3.29	3.44	3.32-3.55	2.91	2.82-3.01	3.02	2.93-3.12
2+ antihypertensives	3.33	3.22-3.44	3.55	3.43-3.66	3.33	3.23-3.44	3.40	3.30-3.51
**RECORDED BP CONTROL**								
1 antihypertensive	1.39	1.33-1.46	1.51	1.44-1.58	1.36	1.29-1.44	1.44	1.38-1.51
2+ antihypertensives	1.37	1.31-1.44	1.41	1.35-1.48	1.28	1.21-1.35	1.42	1.36-1.48

Table S4: Effect of treatment intensity on study outcomes. The reference group (OR = 1.00) are untreated patients (i.e. patient on no antihypertensive medication). **All p-values are <0.001.**

Supplementary Figures

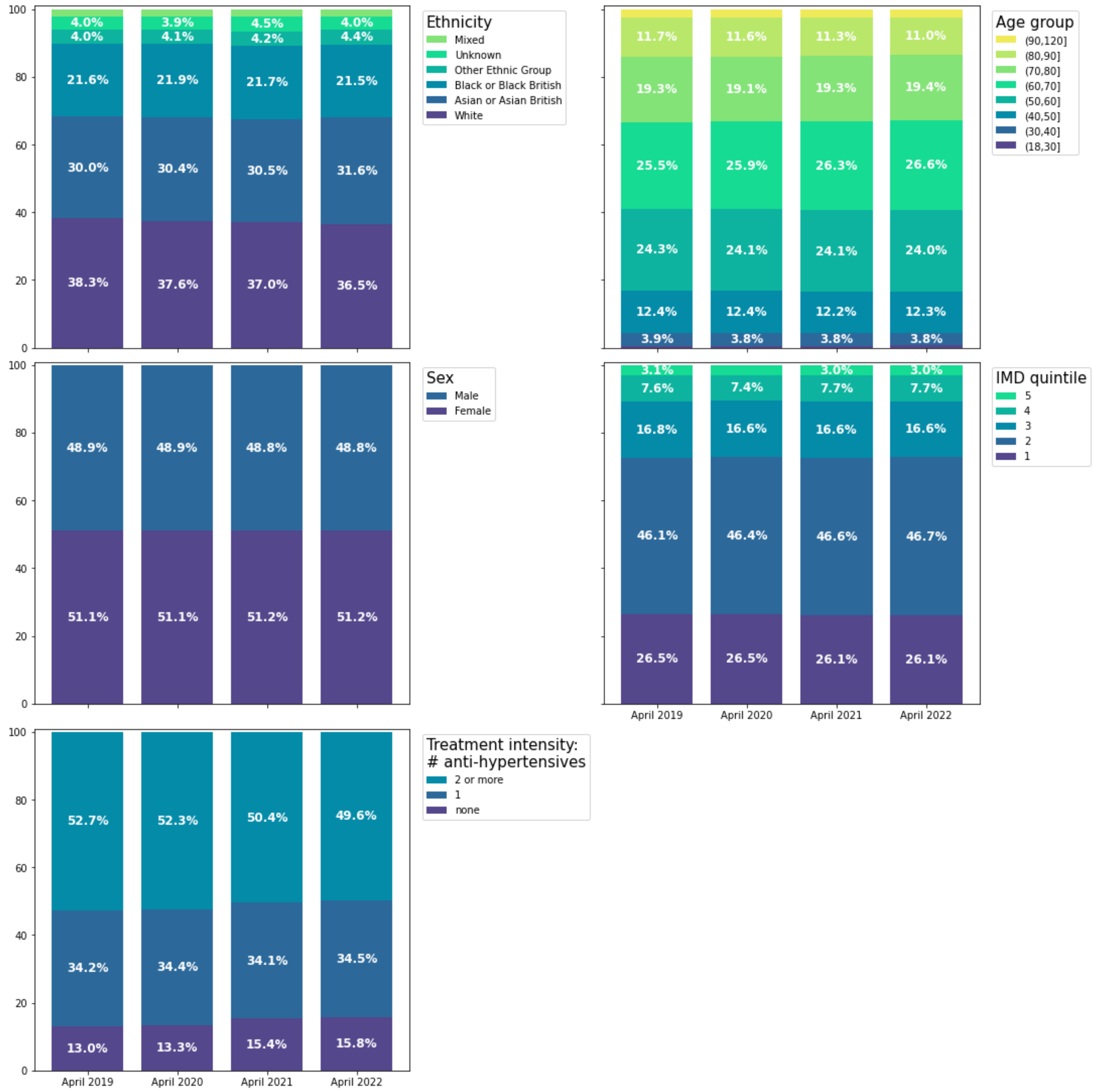

Figure S1: Cohort characteristic (ethnicity, age, sex, IMD quintile and treatment intensity) as a percentage of the cohort for the cohort on 1st April 2019,2020,2021 and 2022). Percentages under 3.0% are not labelled.

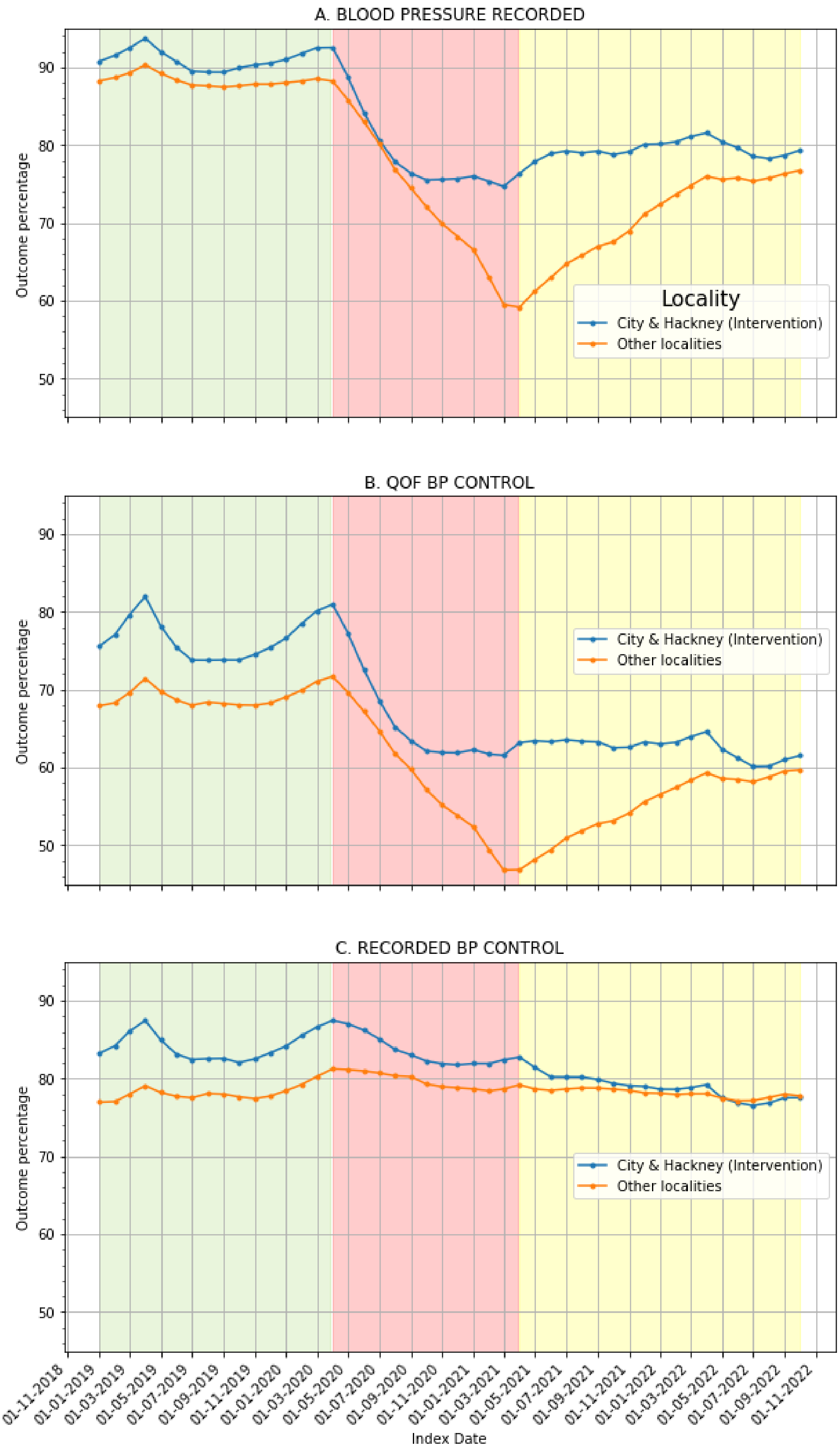

Figure S2: Outcomes (A. BLOOD PRESSURE RECORDED, B. QOF BP CONTROL, C. RECORDED BP CONTROL) by study locality. In May 2020, the City and Hackney locality (blue line) launched a blood pressure recording initiative. Outcomes for the combined non-intervention localities (Newham, Redbridge, Tower Hamlets and Waltham Forest) are plotted with the orange line.

A. BLOOD PRESSURE RECORDED (blood pressure recorded within 12 months of the index date)

Variable	OR (95% Conf. Int.)	P-value
**Asian or Asian British**		
Pre-pandemic (2019)	1.62 (1.55 to 1.71)	<0.001***
Pre-lockdown (2020)	1.58 (1.52 to 1.65)	<0.001***
Lockdown (2021)	1.27 (1.23 to 1.30)	<0.001***
Recovery (2022)	1.39 (1.35 to 1.43)	<0.001***
**Black or Black British**		
Pre-pandemic (2019)	1.09 (1.03 to 1.14)	0.001**
Pre-lockdown (2020)	1.11 (1.06 to 1.16)	<0.001***
Lockdown (2021)	1.07 (1.04 to 1.10)	<0.001***
Recovery (2022)	1.18 (1.14 to 1.22)	<0.001***
**Mixed**		
Pre-pandemic (2019)	0.99 (0.87 to 1.12)	0.85
Pre-lockdown (2020)	1.19 (1.05 to 1.34)	0.005**
Lockdown (2021)	1.05 (0.97 to 1.13)	0.23
Recovery (2022)	1.18 (1.08 to 1.28)	<0.001***
**Other Ethnic Group**		
Pre-pandemic (2019)	1.14 (1.03 to 1.25)	0.008**
Pre-lockdown (2020)	1.18 (1.08 to 1.28)	<0.001***
Lockdown (2021)	1.23 (1.16 to 1.30)	<0.001***
Recovery (2022)	1.15 (1.08 to 1.22)	<0.001***
**Unknown**		
Pre-pandemic (2019)	0.69 (0.64 to 0.74)	<0.001***
Pre-lockdown (2020)	0.78 (0.72 to 0.84)	<0.001***
Lockdown (2021)	0.88 (0.84 to 0.92)	<0.001***
Recovery (2022)	0.77 (0.73 to 0.82)	<0.001***

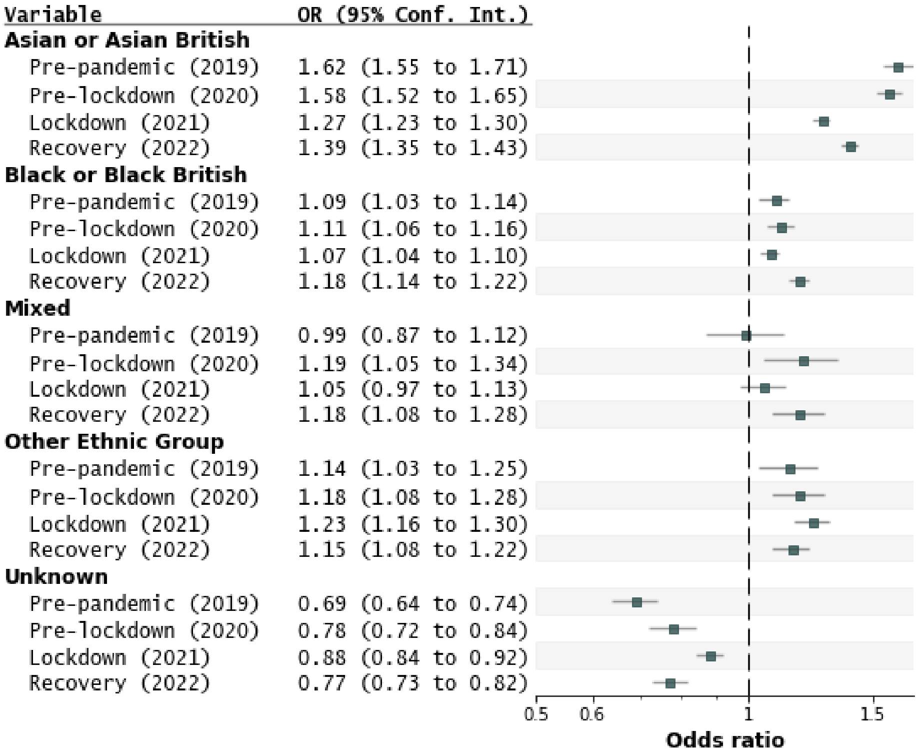

B. QOF BP CONTROL (most recent blood pressure on age-adjusted target; missing blood pressures are deemed uncontrolled)

Variable	OR (95% Conf. Int.)	P-value
**Asian or Asian British**		
Pre-pandemic (2019)	1.39 (1.35 to 1.43)	<0.001***
Pre-lockdown (2020)	1.33 (1.29 to 1.37)	<0.001***
Lockdown (2021)	1.23 (1.20 to 1.26)	<0.001***
Recovery (2022)	1.31 (1.28 to 1.34)	<0.001***
**Black or Black British**		
Pre-pandemic (2019)	0.92 (0.89 to 0.95)	<0.001***
Pre-lockdown (2020)	0.94 (0.91 to 0.97)	<0.001***
Lockdown (2021)	0.96 (0.94 to 0.99)	0.005**
Recovery (2022)	1.00 (0.97 to 1.03)	0.93
**Mixed**		
Pre-pandemic (2019)	0.90 (0.83 to 0.98)	0.01*
Pre-lockdown (2020)	1.03 (0.95 to 1.11)	0.54
Lockdown (2021)	0.99 (0.93 to 1.07)	0.87
Recovery (2022)	0.97 (0.90 to 1.04)	0.35
**Other Ethnic Group**		
Pre-pandemic (2019)	1.26 (1.18 to 1.34)	<0.001***
Pre-lockdown (2020)	1.21 (1.14 to 1.29)	<0.001***
Lockdown (2021)	1.26 (1.20 to 1.33)	<0.001***
Recovery (2022)	1.15 (1.09 to 1.21)	<0.001***
**Unknown**		
Pre-pandemic (2019)	0.75 (0.71 to 0.79)	<0.001***
Pre-lockdown (2020)	0.77 (0.73 to 0.82)	<0.001***
Lockdown (2021)	0.77 (0.74 to 0.81)	<0.001***
Recovery (2022)	0.73 (0.69 to 0.77)	<0.001***

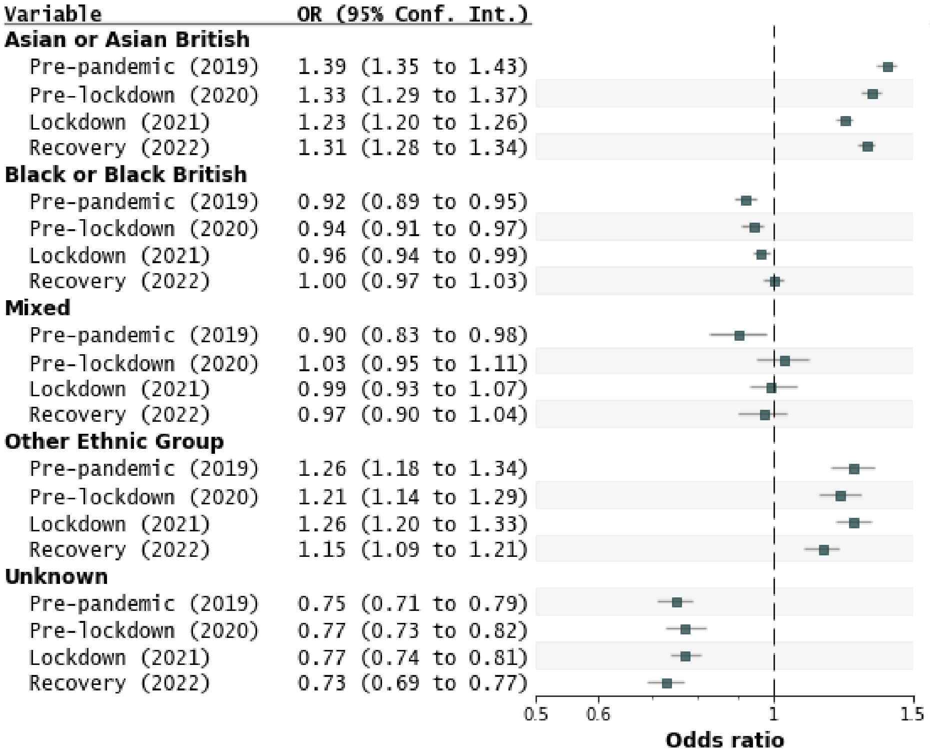

C. RECORDED BP CONTROL (most recent blood pressure on age-adjusted target; only individuals with a blood pressure recorded within last 12 months are considered)

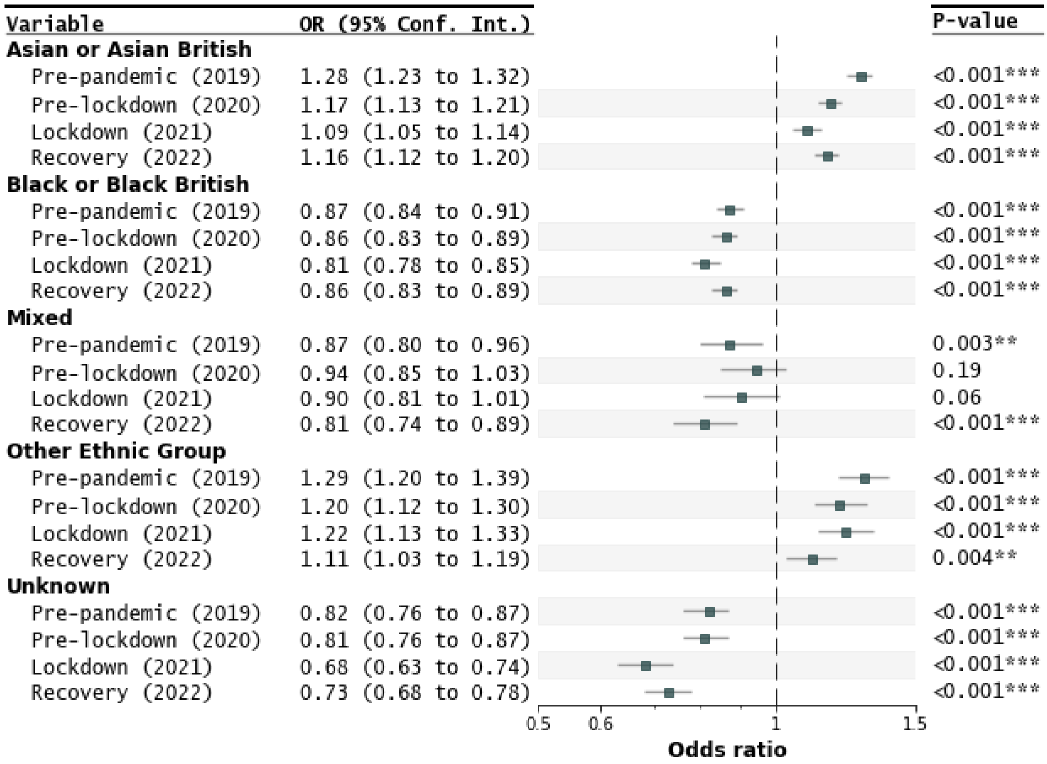

Figure S3: Impact of the pandemic on outcomes of management of hypertension. Forest plots are shown for: A. BLOOD PRESSURE RECORDED, B. QOF BP CONTROL and C. RECORDED BP CONTROL. In each ethnicity group, the reference group is “White” in the same year. The Odds Ratios are derived from a multivariate model adjusted for age, sex, IMD quintile and treatment intensity (see below).

A. **BLOOD PRESSURE RECORDED** (blood pressure recorded within 12 months of the index date)

Variable	OR (95% Conf. Int.)	P-value
**Year**		
Y2020	0.90 (0.82 to 0.98)	0.02*
Y2021	0.31 (0.29 to 0.33)	<0.001***
Y2022	0.51 (0.48 to 0.55)	<0.001***
**Year : Ethnicity**		
2022:Unknown	0.77 (0.73 to 0.82)	<0.001***
2021:Unknown	0.88 (0.84 to 0.92)	<0.001***
2020:Unknown	0.78 (0.72 to 0.84)	<0.001***
2019:Unknown	0.69 (0.64 to 0.74)	<0.001***
2022:Other Ethnic Group	1.15 (1.08 to 1.22)	<0.001***
2020:Other Ethnic Group	1.18 (1.08 to 1.28)	<0.001***
2019:Other Ethnic Group	1.14 (1.03 to 1.25)	0.008**
2022:Mixed	1.18 (1.08 to 1.28)	<0.001***
2021:Mixed	1.05 (0.97 to 1.13)	0.23
2021:Other Ethnic Group	1.23 (1.16 to 1.30)	<0.001***
2019:Mixed	0.99 (0.87 to 1.12)	0.85
2022:Black or Black British	1.18 (1.14 to 1.22)	<0.001***
2021:Black or Black British	1.07 (1.04 to 1.10)	<0.001***
2020:Black or Black British	1.11 (1.06 to 1.16)	<0.001***
2019:Black or Black British	1.09 (1.03 to 1.14)	0.001**
2022:Asian or Asian British	1.39 (1.35 to 1.43)	<0.001***
2021:Asian or Asian British	1.27 (1.23 to 1.30)	<0.001***
2020:Asian or Asian British	1.58 (1.52 to 1.65)	<0.001***
2019:Asian or Asian British	1.62 (1.55 to 1.71)	<0.001***
2020:Mixed	1.19 (1.05 to 1.34)	0.005**
**Year : Age**		
2019:(70,80]	1.25 (1.18 to 1.33)	<0.001***
2020:(70,80]	1.14 (1.08 to 1.20)	<0.001***
2022:(70,80]	1.14 (1.10 to 1.19)	<0.001***
2019:(80,90]	1.37 (1.27 to 1.48)	<0.001***
2020:(80,90]	1.13 (1.06 to 1.21)	<0.001***
2021:(90,120]	1.24 (1.15 to 1.35)	<0.001***
2022:(80,90]	1.15 (1.09 to 1.20)	<0.001***
2019:(90,120]	1.37 (1.18 to 1.60)	<0.001***
2020:(90,120]	1.27 (1.11 to 1.46)	<0.001***
2022:(90,120]	1.10 (1.00 to 1.20)	0.05*
2022:(50,60]	0.85 (0.82 to 0.87)	<0.001***
2021:(80,90]	1.11 (1.07 to 1.15)	<0.001***
2021:(50,60]	0.88 (0.86 to 0.91)	<0.001***
2021:(70,80]	1.09 (1.05 to 1.12)	<0.001***
2019:(50,60]	0.82 (0.78 to 0.86)	<0.001***
2020:(50,60]	0.84 (0.80 to 0.88)	<0.001***
2020:(17,30]	0.90 (0.78 to 1.05)	0.18
2021:(17,30]	1.16 (1.03 to 1.31)	0.01*
2022:(17,30]	0.92 (0.82 to 1.04)	0.19
2019:(30,40]	0.79 (0.73 to 0.86)	<0.001***
2020:(30,40]	0.83 (0.77 to 0.89)	<0.001***
2019:(17,30]	1.07 (0.91 to 1.27)	0.43
2022:(30,40]	0.87 (0.82 to 0.93)	<0.001***
2019:(40,50]	0.81 (0.76 to 0.86)	<0.001***
2022:(40,50]	0.85 (0.82 to 0.89)	<0.001***
2020:(40,50]	0.82 (0.77 to 0.86)	<0.001***
2021:(40,50]	0.89 (0.86 to 0.93)	<0.001***
2021:(30,40]	0.96 (0.91 to 1.02)	0.17
**Year : Sex**		
2021:Female	1.08 (1.06 to 1.11)	<0.001***
2022:Female	1.13 (1.10 to 1.16)	<0.001***
2019:Female	1.30 (1.26 to 1.35)	<0.001***
2020:Female	1.25 (1.21 to 1.30)	<0.001***
**Year : IMD quintile**		
2022:Q3	0.71 (0.68 to 0.74)	<0.001***
2021:Q5	0.67 (0.63 to 0.71)	<0.001***
2020:Q5	0.72 (0.65 to 0.79)	<0.001***
2019:Q5	0.69 (0.62 to 0.77)	<0.001***
2022:Q4	0.67 (0.64 to 0.70)	<0.001***
2022:Q5	0.70 (0.65 to 0.75)	<0.001***
2021:Q4	0.65 (0.62 to 0.68)	<0.001***
2020:Q4	0.64 (0.60 to 0.69)	<0.001***
2019:Q4	0.65 (0.61 to 0.70)	<0.001***
2021:Q3	0.72 (0.69 to 0.74)	<0.001***
2019:Q2	0.90 (0.86 to 0.94)	<0.001***
2019:Q3	0.73 (0.69 to 0.77)	<0.001***
2022:Q2	0.99 (0.96 to 1.02)	0.34
2021:Q2	0.93 (0.90 to 0.95)	<0.001***
2020:Q2	0.88 (0.84 to 0.92)	<0.001***
2022:Q0	0.94 (0.50 to 1.81)	0.84
2021:Q0	0.64 (0.37 to 1.10)	0.1
2020:Q0	0.74 (0.38 to 1.55)	0.4
2019:Q0	0.66 (0.32 to 1.48)	0.28
2020:Q3	0.75 (0.71 to 0.79)	<0.001***
**Year : Treatment intensity**		
2020:2Plus	8.57 (8.23 to 8.93)	<0.001***
2019:2Plus	9.99 (9.55 to 10.45)	<0.001***
2022:1	4.05 (3.93 to 4.19)	<0.001***
2021:2Plus	4.10 (3.97 to 4.22)	<0.001***
2020:1	6.72 (6.44 to 7.01)	<0.001***
2019:1	7.83 (7.48 to 8.21)	<0.001***
2021:1	3.27 (3.17 to 3.38)	<0.001***
2022:2Plus	5.18 (5.02 to 5.35)	<0.001***

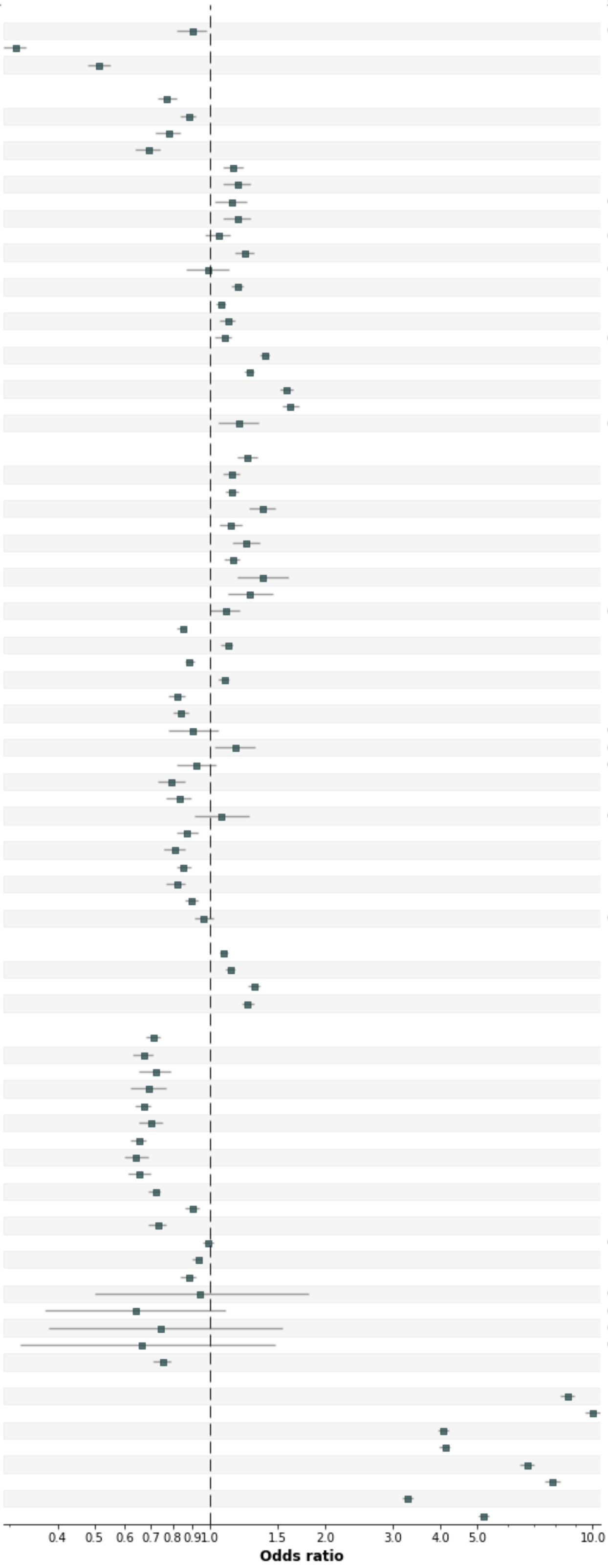

B. QOF BP CONTROL (most recent blood pressure on age-adjusted target; missing blood pressures are deemed uncontrolled)

Variable	OR (95% Conf. Int.)	P-value
**Year**		
Y2020	0.98 (0.92 to 1.04)	0.46
Y2021	0.38 (0.36 to 0.41)	<0.001***
Y2022	0.53 (0.50 to 0.57)	<0.001***
**Year : Ethnicity**		
2022:Unknown	0.73 (0.69 to 0.77)	<0.001***
2021:Unknown	0.77 (0.74 to 0.81)	<0.001***
2020:Unknown	0.77 (0.73 to 0.82)	<0.001***
2019:Unknown	0.75 (0.71 to 0.79)	<0.001***
2022:Other Ethnic Group	1.15 (1.09 to 1.21)	<0.001***
2020:Other Ethnic Group	1.21 (1.14 to 1.29)	<0.001***
2019:Other Ethnic Group	1.26 (1.18 to 1.34)	<0.001***
2022:Mixed	0.97 (0.90 to 1.04)	0.35
2021:Mixed	0.99 (0.93 to 1.07)	0.87
2021:Other Ethnic Group	1.26 (1.20 to 1.33)	<0.001***
2019:Mixed	0.90 (0.83 to 0.98)	0.01*
2022:Black or Black British	1.00 (0.97 to 1.03)	0.93
2021:Black or Black British	0.96 (0.94 to 0.99)	0.005**
2020:Black or Black British	0.94 (0.91 to 0.97)	<0.001***
2019:Black or Black British	0.92 (0.89 to 0.95)	<0.001***
2022:Asian or Asian British	1.31 (1.28 to 1.34)	<0.001***
2021:Asian or Asian British	1.23 (1.20 to 1.26)	<0.001***
2020:Asian or Asian British	1.33 (1.29 to 1.37)	<0.001***
2019:Asian or Asian British	1.39 (1.35 to 1.43)	<0.001***
2020:Mixed	1.03 (0.95 to 1.11)	0.54
**Year : Age**		
2019:(70,80]	1.13 (1.09 to 1.17)	<0.001***
2020:(70,80]	1.14 (1.10 to 1.19)	<0.001***
2022:(70,80]	1.14 (1.11 to 1.18)	<0.001***
2019:(80,90]	2.25 (2.14 to 2.37)	<0.001***
2020:(80,90]	1.69 (1.61 to 1.77)	<0.001***
2021:(90,120]	1.33 (1.23 to 1.43)	<0.001***
2022:(80,90]	1.50 (1.44 to 1.56)	<0.001***
2019:(90,120]	2.02 (1.82 to 2.24)	<0.001***
2020:(90,120]	1.50 (1.36 to 1.65)	<0.001***
2022:(90,120]	1.32 (1.22 to 1.43)	<0.001***
2022:(50,60]	0.81 (0.78 to 0.83)	<0.001***
2021:(80,90]	1.30 (1.26 to 1.35)	<0.001***
2021:(50,60]	0.84 (0.82 to 0.87)	<0.001***
2021:(70,80]	1.06 (1.03 to 1.10)	<0.001***
2019:(50,60]	0.82 (0.79 to 0.85)	<0.001***
2020:(50,60]	0.78 (0.76 to 0.81)	<0.001***
2020:(17,30]	0.70 (0.62 to 0.79)	<0.001***
2021:(17,30]	0.95 (0.84 to 1.07)	0.4
2022:(17,30]	0.69 (0.61 to 0.77)	<0.001***
2019:(30,40]	0.64 (0.60 to 0.68)	<0.001***
2020:(30,40]	0.57 (0.54 to 0.60)	<0.001***
2019:(17,30]	0.65 (0.57 to 0.74)	<0.001***
2022:(30,40]	0.64 (0.61 to 0.68)	<0.001***
2019:(40,50]	0.70 (0.67 to 0.72)	<0.001***
2022:(40,50]	0.70 (0.68 to 0.73)	<0.001***
2020:(40,50]	0.65 (0.62 to 0.67)	<0.001***
2021:(40,50]	0.77 (0.74 to 0.80)	<0.001***
2021:(30,40]	0.74 (0.70 to 0.78)	<0.001***
**Year : Sex**		
2021:Female	1.07 (1.05 to 1.09)	<0.001***
2022:Female	1.12 (1.10 to 1.14)	<0.001***
2019:Female	1.19 (1.16 to 1.22)	<0.001***
2020:Female	1.19 (1.16 to 1.21)	<0.001***
**Year : IMD quintile**		
2022:Q3	0.80 (0.77 to 0.82)	<0.001***
2021:Q5	0.73 (0.69 to 0.78)	<0.001***
2020:Q5	0.72 (0.67 to 0.77)	<0.001***
2019:Q5	0.70 (0.65 to 0.75)	<0.001***
2022:Q4	0.82 (0.79 to 0.86)	<0.001***
2022:Q5	0.84 (0.79 to 0.89)	<0.001***
2021:Q4	0.72 (0.69 to 0.75)	<0.001***
2020:Q4	0.73 (0.70 to 0.77)	<0.001***
2019:Q4	0.68 (0.65 to 0.71)	<0.001***
2021:Q3	0.75 (0.73 to 0.78)	<0.001***
2019:Q2	0.88 (0.86 to 0.91)	<0.001***
2019:Q3	0.72 (0.69 to 0.74)	<0.001***
2022:Q2	1.00 (0.98 to 1.03)	0.92
2021:Q2	0.92 (0.90 to 0.95)	<0.001***
2020:Q2	0.89 (0.87 to 0.92)	<0.001***
2022:Q0	0.76 (0.43 to 1.36)	0.36
2021:Q0	0.64 (0.36 to 1.12)	0.13
2020:Q0	0.65 (0.39 to 1.12)	0.11
2019:Q0	0.82 (0.47 to 1.47)	0.49
2020:Q3	0.79 (0.76 to 0.82)	<0.001***
**Year : Treatment intensity**		
2020:2Plus	3.55 (3.43 to 3.66)	<0.001***
2019:2Plus	3.33 (3.22 to 3.44)	<0.001***
2022:1	3.02 (2.93 to 3.12)	<0.001***
2021:2Plus	3.33 (3.23 to 3.44)	<0.001***
2020:1	3.44 (3.32 to 3.55)	<0.001***
2019:1	3.17 (3.07 to 3.29)	<0.001***
2021:1	2.91 (2.82 to 3.01)	<0.001***
2022:2Plus	3.40 (3.30 to 3.51)	<0.001***

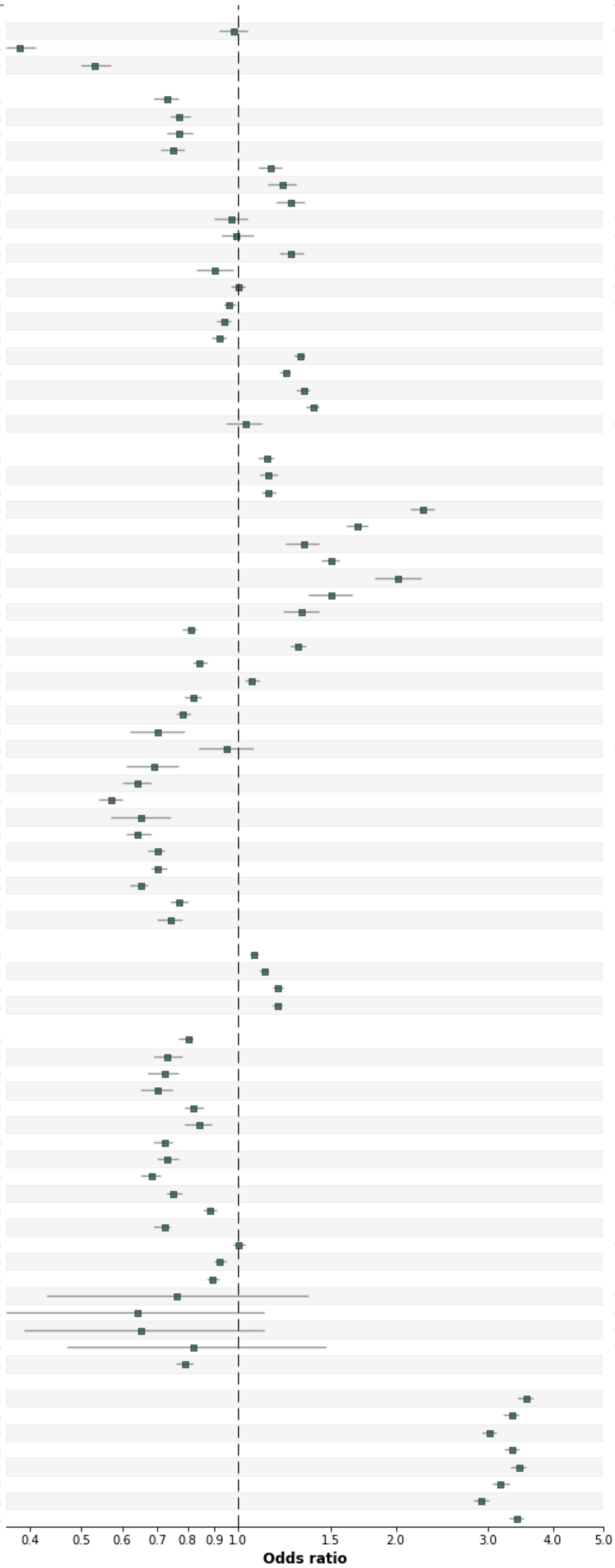

C. RECORDED BP CONTROL (most recent blood pressure on age-adjusted target; only individuals with a blood pressure recorded within last 12 months are considered)

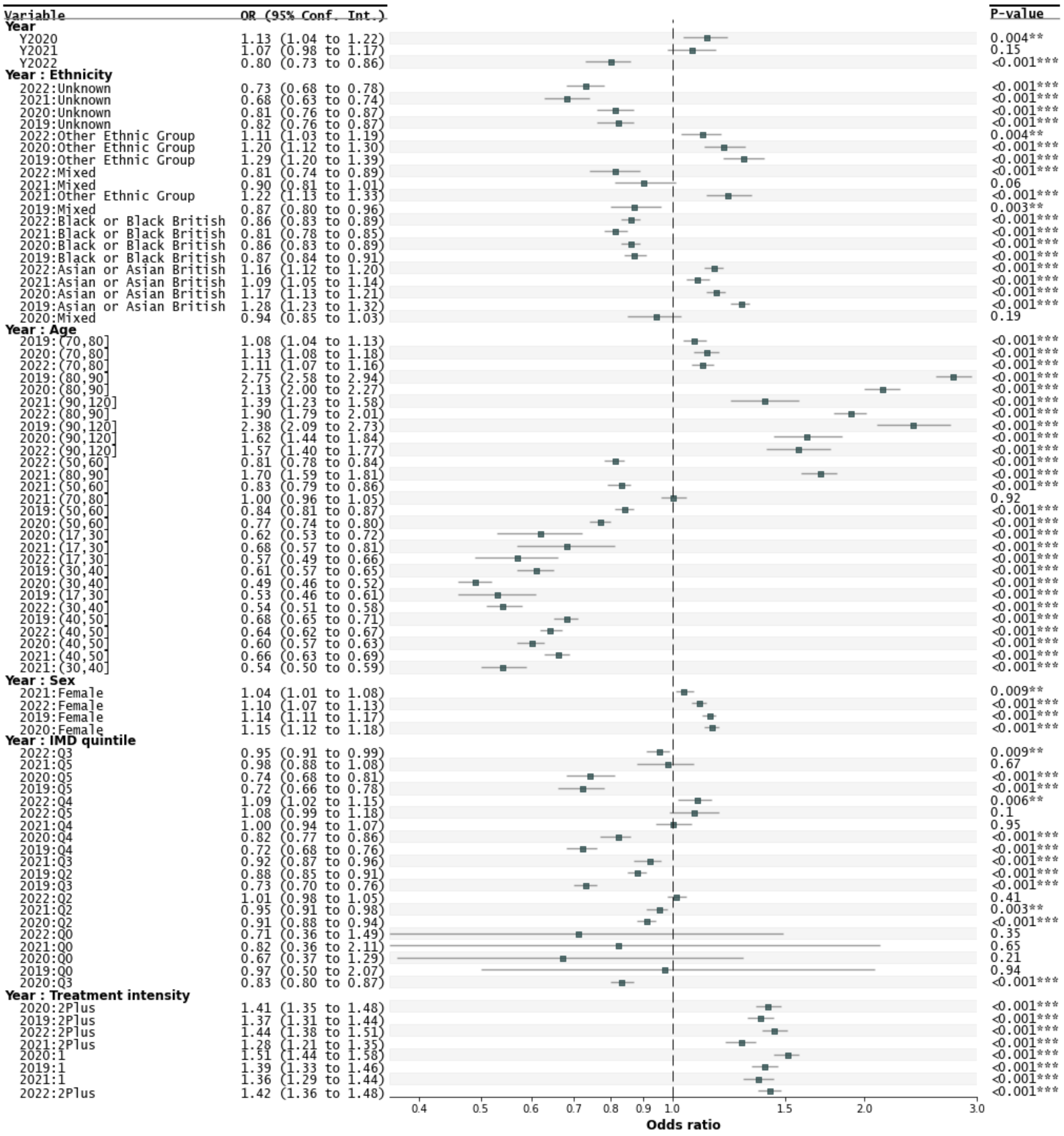

Figure S4: Full data for multivariate analysis with years for A. BLOOD PRESSURE RECORDED, B. QOF BP CONTROL and C. RECORDED BP CONTROL. *: p-val <0.05; **: p-val<0.01; ***: p-val<0.001.
