# Supplementary material for: COVID-19 pandemic impact on hypertension management in North East London: an observational cohort study using electronic health records
Source: BMJ Open. 2024 Aug 6;14(8):e083497. doi: 10.1136/bmjopen-2023-083497 (PMC11308888; doi:10.1136/bmjopen-2023-083497)
Supplement: Supplementary file 2 [file bmjopen-2023-083497.reviewer_comments.pdf]

PEER REVIEW HISTORY

BMJ Open publishes all reviews undertaken for accepted manuscripts. Reviewers are asked to complete a checklist review form (**http://bmjopen.bmj.com/site/about/resources/checklist.pdf**) and are provided with free text boxes to elaborate on their assessment. These free text comments are reproduced below.

ARTICLE DETAILS

**TITLE (PROVISIONAL)**	COVID-19 pandemic impact on hypertension management in North-East London: an observational cohort study using electronic health records.
**AUTHORS**	Rison, Stuart; Redfern, Oliver; Mathur, Rohini; Dostal, Isabel; Carvalho, Chris; Raisi-Estabragh, Zahra; Robson, John

VERSION 1 – REVIEW

**REVIEWER**	Adjaye-Gbewonyo, Kafui<br>University of Greenwich, Faculty of Education, Health and Human Sciences
**REVIEW RETURNED**	08-Mar-2024

**GENERAL COMMENTS**	Overall, this is a well-written manuscript examining the potential effects of the COVID-19 pandemic on hypertension management and control in Northeast London. The information presented is relatively clear and accompanied by detailed tables and figures. I recommend a few minor revisions and corrections to improve clarity of the reporting. These are described below:<br><br>**Abstract**:<br>• *Results*: The last line of the first paragraph of the results might benefit from rewording to clarify. The meaning of this statement becomes clear in the body of the paper, but for readers who are screening or reading only the abstract, it may be interpreted to mean different things. For example, an alternative way of wording it might be, 'However, when excluding individuals without a recent blood pressure recording...'<br><br>**Strengths and limitations**<br>• *Bullet point 3*: The first sentence of the third strength and limitation might also benefit from rewording for clarity.<br><br>**Introduction**<br>• *First paragraph, second sentence*: This could be corrected to read, '…transitioned from largely face-to-face primary care appointment models to remote-consultation models <u>leading to</u> reduced direct access opportunities.' (The 'of' should be deleted.)<br>• *Second paragraph, first sentence*: Could the phrase 'ethnic groups' be expressed as 'demographic groups' to reflect some of the broader inequalities observed in SARS-CoV-2 infection and mortality rates?<br><br>**Methods**<br>• *Statistical Methods, second paragraph, first sentence*: I might reword this sentence as follows for clarity: 'Unadjusted and

	adjusted (for ethnic group, sex, age, IMD quintile and treatment intensity) multivariable logistic regression analyses were completed for each outcome indicator.'<br><br>**Results**<br>• *Outcomes, second paragraph*: The p-value reported for the correlation between BLOOD_PRESSURE_RECORDED and BLOOD_PRESSURE_CONTROLLED appears to be incorrect. The p-value is reported as 3.90, so this appears to be another statistic (e.g. t statistic) rather than a probability.<br>• *Study phases, Pre-pandemic phase analyses*: Could a citation be included in the first line to reference the previously published work?<br>• *Variance between study period phases – ethnic variations, paragraph 2*: In the first sentence, rather than saying that the Black ethnic group outcomes were 'worse', could this result be described more fully? For example, they 'had lower odds of having controlled hypertension and controlled blood pressure'. Could the last sentence of that paragraph also be reworded (e.g., 'Conversely, the Asian ethnic group always had higher odds of the outcomes than the White ethnic group.')?<br>• *Variance between study period phases – ethnic variations, paragraph 3*: Again, could the result presented in the first sentence be described in more detail? (E.g., 'For the BLOOD_PRESSURE_RECORDED indicator, the Asian ethnic group had higher odds at all time points.')?<br><br>**Discussion**<br>• Is there scope within the Discussion section to briefly mention how some other study characteristics such as the sample size might affect the interpretation of the results as a strength or limitation?<br>• *Comparison with existing literature*: There is a typo in the last sentence where the 'L' was accidentally left out of the indicator name BLOOD_PRESSURE_CONTROL.<br><br>**Conclusions**<br>• *Second paragraph, first sentence*: I might suggest mentioning males again as this is a group that may also warrant targeted intervention.

**REVIEWER**	Kumi, Dennis<br>John H Stroger Jr Hospital of Cook County, Department of Medicine
**REVIEW RETURNED**	17-Apr-2024

**GENERAL COMMENTS**	ABOUT THE WRITING OF THE MANUSCRIPT<br><br>GENERAL COMMENTS<br>The writing style is very hard to follow<br>Too many abbreviations, most of which are colloquial and some are no pre-defined<br>Some parts of the manuscript do not follow usual style of medical literature<br>Some outcomes are poorly defined.<br>I suggest the writer rewrites the paper for resubmission<br>The idea is quite exciting and it seems a lot of great work has gone into this but it has been poorly written unfortunately.<br><br>THE METHOD

	It was not well described overall.<br>Especially the outcome variables were ambiguous such as HYPERTENSION_CONTROLLED vs BLOOD PRESSURE_CONTROLLED.<br>There were details about the statistical analysis such as using interaction terms in the software to obtain the outcome measures. These are not useful to the reader.<br>How data was treated such as categorical vs continuous data was not described.<br>The author compared the trajectory of the graphs for the various outcomes using corellation coefficient. It is not clear what analysis he did with individual graph patterns themselves . Within the various timelines as described, it is not clear if the author performed a trend analysis, time to event analysis of an open cohort, estimated hazard ratios etc.<br><br>RESULTS<br>Initial statements in results section was further describing the study population and setting of study which was not appropriately placed.<br>Parts of the results also sought to talk about another study, possibly a substudy not previously described in the method and that was quite unexpected.<br>Other parts of the results also tries to offer explanations to the findings of the study and mentions facts from literature in support and these are not appropriately placed.<br><br>STUDY LIMITATIONS<br>The study limitations talks of information better suited for discussion.<br>The study limitation talks of two major confounders that are not addressed by the methodology or design and thus makes the conclusion of the study hard to accept.

VERSION 1 – AUTHOR RESPONSE

Reviewer: 1
Dr. Kafui Adjaye-Gbewonyo, University of Greenwich
Comments to the Author:
Overall, this is a well-written manuscript examining the potential effects of the COVID-19 pandemic on hypertension management and control in Northeast London. The information presented is relatively clear and accompanied by detailed tables and figures. I recommend a few minor revisions and corrections to improve clarity of the reporting. These are described below:

Abstract:
• *Results*: The last line of the first paragraph of the results might benefit from rewording to clarify. The meaning of this statement becomes clear in the body of the paper, but for readers who are screening or reading only the abstract, it may be interpreted to mean different things. For example, an alternative way of wording it might be, 'However, when excluding individuals without a recent blood pressure recording...'

We have edited the abstract and modified this sentence to "Excluding patients without a recent blood pressure recording, the indicators increased to 81%, 80% and 78% respectively."

Strengths and limitations
• *Bullet point 3*: The first sentence of the third strength and limitation might also benefit from rewording for clarity.

We have removed this bullet point (now discussed in the Limitations section of the manuscript).

Introduction
• *First paragraph, second sentence*: This could be corrected to read, '…transitioned from largely face-to-face primary care appointment models to remote-consultation models leading to reduced direct access opportunities.' (The 'of' should be deleted.)

Thank you, this has been corrected.

• *Second paragraph, first sentence*: Could the phrase 'ethnic groups' be expressed as 'demographic groups' to reflect some of the broader inequalities observed in SARS-CoV-2 infection and mortality rates?

Changed as suggested.

Methods
• *Statistical Methods, second paragraph, first sentence*: I might reword this sentence as follows for clarity: 'Unadjusted and adjusted (for ethnic group, sex, age, IMD quintile and treatment intensity) multivariable logistic regression analyses were completed for each outcome indicator.'

Changed as suggested.

Results
• *Outcomes, second paragraph*: The p-value reported for the correlation between BLOOD_PRESSURE_RECORDED and BLOOD_PRESSURE_CONTROLLED appears to be incorrect. The p-value is reported as 3.90, so this appears to be another statistic (e.g. t statistic) rather than a probability.

Thanks for highlighting this. We have recalculated correlations using Kendall's Tau test and updated all p-values accordingly.

• *Study phases, Pre-pandemic phase analyses*: Could a citation be included in the first line to reference the previously published work?

We have modified the results section according to reviewer 2's suggestion. Our previous study is now referenced in both the Introduction and the Discussion.

• *Variance between study period phases – ethnic variations, paragraph 2*: In the first sentence, rather than saying that the Black ethnic group outcomes were 'worse', could this result be described more fully? For example, they 'had lower odds of having controlled hypertension and controlled blood pressure'. Could the last sentence of that paragraph also be reworded (e.g., 'Conversely, the Asian ethnic group always had higher odds of the outcomes than the White ethnic group.')?
• *Variance between study period phases – ethnic variations, paragraph 3*: Again, could the result presented in the first sentence be described in more detail? (E.g., 'For the

BLOOD_PRESSURE_RECORDED indicator, the Asian ethnic group had higher odds at all time points.')?

This section has been amended including the above suggested changes.

Discussion
• Is there scope within the Discussion section to briefly mention how some other study characteristics such as the sample size might affect the interpretation of the results as a strength or limitation?

We discuss this in the amended Discussion and the new Limitations section.

• *Comparison with existing literature*: There is a typo in the last sentence where the 'L' was accidentally left out of the indicator name BLOOD_PRESSURE_CONTROL.

In line with reviewer 2's comments, we have altered the outcome names.

Conclusions
• *Second paragraph, first sentence*: I might suggest mentioning males again as this is a group that may also warrant targeted intervention.

Done.

<u>Reviewer: 2</u>

Dr. Dennis Kumi, John H Stroger Jr Hospital of Cook County

Comments to the Author:

ABOUT THE WRITING OF THE MANUSCRIPT

GENERAL COMMENTS

The writing style is very hard to follow

We have redrafted the manuscript in light of comments from both reviewers.

Too many abbreviations, most of which are colloquial and some are no pre-defined.

We have reduced the number abbreviation and pre-defined these. We appreciate that standard terminology widely used in the UK (e.g. QOF indicators. IMD) may be less familiar to an international audience. We have clarified these and made sure all are appropriately referenced.

Some parts of the manuscript do not follow usual style of medical literature

We have redrafted the manuscript and ensured that we conform to scientific writing standards in line with journal specifications.

Some outcomes are poorly defined.

We have modified the outcomes section to communicate more clearly the definition of each outcome.

I suggest the writer rewrites the paper for resubmission

The idea is quite exciting and it seems a lot of great work has gone into this but it has been poorly written unfortunately.

We hope that the modified manuscript addresses the second reviewer's concerns.

THE METHOD

It was not well described overall.

Especially the outcome variables were ambiguous such as HYPERTENSION_CONTROLLED vs BLOOD PRESSURE_CONTROLLED.

We have modified the names of the outcomes to: QOF BP CONTROL and RECORDED BP CONTROLLED. These terms better reflect the underlying rationale for each outcome, namely that the former is based on the NHS England's QOF criteria, and the latter only considers patients for whom a blood pressure was recorded. We have added a paragraph clarifying the difference between these two outcomes.

There were details about the statistical analysis such as using interaction terms in the software to obtain the outcome measures. These are not useful to the reader.

We have modified the Methods section to remove unnecessary information.

How data was treated such as categorical vs continuous data was not described.

All data were categorical. Sex (M, F), Age ((18,30], (30,40], (40,50], (50,60], (70,80], (80,90], (90,120]), Ethnicity (Asian, Black, White, Mixed, Other, Unknown), IMD quintile (1,2,3,4,5), Study phase (2019, 2020, 2021 and 2022) and treatment intensity (untreated, single antihypertensive, 2+ antihypertensives). This had been clarified in the methods section.

The author compared the trajectory of the graphs for the various outcomes using corellation coefficient. It is not clear what analysis he did with individual graph patterns themselves . Within the various timelines as described, it is not clear if the author performed a trend analysis, time to event analysis of an open cohort, estimated hazard ratios etc.

We have amended the manuscript to clarify that we plotted the percentage of cohort individuals meeting each outcome criteria against each study month. We performed pairwise correlations of the outcomes (BLOOD PRESSURE RECORDED and QOF BP CONTROL; BLOOD PRESSURE RECORDED and RECORDED BP CONTROL). In reviewing the manuscript, we noted that the previously used Pearson correlation analysis performed was not most appropriate and we therefore recalculated outcome correlations using Kendall's rank correlation coefficient (Kendall's τ). This change did not impact the correlation conclusions, i.e. that the recording of blood pressures had a noticeable impact on the QOF BP CONTROL, but not on the RECORDED BP CONTROL.

RESULTS

Initial statements in results section was further describing the study population and setting of study which was not appropriately placed.

We have moved the paragraph to the Methods (study cohort) section of the manuscript.

Parts of the results also sought to talk about another study, possibly a substudy not previously described in the method and that was quite unexpected.

We now clarify that this study develops a previous study in the Introduction and in the Discussion. We have re-written the results section.

Other parts of the results also tries to offer explanations to the findings of the study and mentions facts from literature in support and these are not appropriately placed.

We have re-written the results section.

STUDY LIMITATIONS

The study limitations talks of information better suited for discussion.

We have edited the "STRENGTHS AND LIMITATIONS" section in line with the editor's and this reviewer's comments.

The study limitation talks of two major confounders that are not addressed by the methodology or design and thus makes the conclusion of the study hard to accept.

We have added a Limitation section to the manuscript. No study of long-term conditions could accommodate all possible cofounders. For example, the stated exemplar confounders (medication adherence and possible physiological ethnic group differences) are not considered in references 28, 29, 30, 33 and 34 cited in our manuscript, without invalidating their findings.

VERSION 2 – REVIEW

**REVIEWER**	Kumi, Dennis<br>John H Stroger Jr Hospital of Cook County, Department of Medicine
**REVIEW RETURNED**	12-Jun-2024

**GENERAL COMMENTS**	The author and colleges have done a great job and have improved on prior submision as well. Comments for correction or explanation are as below;<br><br>ABSTRACT<br>. The conclusion sounded a bit misleading. The author concluded that " : The COVID-19 pandemic had a greater impact on blood pressure recording than on<br>blood pressure control" . Though this is obvious from reading your results and discussions, the impact on blood pressure recording was not mentioned anywhere in your abstract. Many readers may only read your abstract thus it must have concise but with the complete information leading to this conclusion.<br><br>DISCUSSION<br>On line 47 to 49, statements are made in reference to results from another database or study. Kindly cite references in text to support.<br><br>CONCLUSION<br>The conclusion should have a a clear statement which is summary of your major findings. For a reader who simply skims to read the conclusion of your study, they should get a summary of the study outcomes. The author talked mainly about the relevance of the

	study findings which is acceptable but should not replace the concluding statements of facts.

VERSION 2 – AUTHOR RESPONSE

Reviewer: 2

Dr. Dennis Kumi, John H Stroger Jr Hospital of Cook County

Comments to the Author:

The author and colleges have done a great job and have improved on prior submision as well. Comments for correction or explanation are as below;

We are grateful to Dr Kumi for his valuable review and the positive impact it had on our re-submission.

ABSTRACT

. The conclusion sounded a bit misleading. The author concluded that " : The COVID-19 pandemic had a greater impact on blood pressure recording than on

blood pressure control" . Though this is obvious from reading your results and discussions, the impact on blood pressure recording was not mentioned anywhere in your abstract. Many readers may only read your abstract thus it must have concise but with the complete information leading to this conclusion.

We have modified the abstract to include a reference to the fall in recorded blood pressures during the pandemic and modified the abstract slightly to account for the abstract conclusion.

Results: *The proportion of patients with a contemporaneous blood pressure recording fell from a 91% pre-pandemic peak to 62% at the end of the pandemic lock-down and improved to 77% by the end of the study. This was paralleled by the proportion of individuals with controlled hypertension which fell from a 73% pre-pandemic peak to 50% at the end of the pandemic lock-down, and improved to 60% by the end of the study. However, when excluding patients without a recent blood pressure recording, the proportions of patients with controlled hypertension increased to 81%, 80% and 78% respectively.*

DISCUSSION

On line 47 to 49, statements are made in reference to results from another database or study. Kindly cite references in text to support.

Citations added.

CONCLUSION

The conclusion should have a a clear statement which is summary of your major findings. For a reader who simply skims to read the conclusion of your study, they should get a summary of the study outcomes. The author talked mainly about the relevance of the study findings which is acceptable but should not replace the concluding statements of facts.

We have modified the conclusion to include a clear summary statement.

Pre-existing inequities in blood pressure monitoring and control persisted during the pandemic and remain outstanding. In the study cohort, the COVID-19 pandemic had a greater impact on blood pressure recording than on blood pressure control per se.

Metrics of hypertension management which combine aspects of monitoring and control of blood pressure might therefore be prone to misinterpretation. QOF indicators may serve as a suitable "performance" metrics, but a full assessment of hypertension control requires separate consideration of both the recording of blood pressure and adequate control in those recorded.

The COVID-19 pandemic effected a radical change in the provision of primary care services and reduced opportunities for recording of risk factors and optimising management. Our data show that despite substantial improvements, more than a year after the pandemic, recovery to pre-pandemic levels had yet to be achieved. Equitable improvements in the management of hypertension remained an outstanding priority for men, younger individuals, and within the Black and Mixed ethnic groups.(44) Furthermore, the lack of ethnicity recording may alert clinicians to individuals at a higher risk of sub-optimal care.
